# Probing the unfolded protein response in long-lived naked mole-rats

**DOI:** 10.1016/j.bbrc.2020.06.118

**Published:** 2020-09-03

**Authors:** Zhen Du, Sampurna Chakrabarti, Yavuz Kulaberoglu, Ewan St John Smith, Christopher M. Dobson, Laura S. Itzhaki, Janet R. Kumita

**Affiliations:** a Department of Pharmacology, University of Cambridge, Tennis Court Rd., Cambridge, CB2 1PD, UK; b Centre for Misfolding Diseases, Department of Chemistry, University of Cambridge, Lensfield Rd., Cambridge, CB2 1EW, UK; c UCL Institute of Healthy Ageing, Darwin Building, 104 Gower St, Bloomsbury, London, WC1E 6AD, UK

**Keywords:** Naked mole-rat, Unfolded protein response, ER stress, Protein homeostasis, Ageing, **NMR**, naked mole-rat, **UPR**, unfolded protein response, **IRE1**, inositol-requiring enzyme 1, **PERK**, protein kinase R-like ER kinase, **ATF6**, activating transcription factor 6, **XBP1**, X-box-binding protein 1, **RIDD**, regulated IRE1-dependent decay, **ERAD**, ER-associated degradation, **CHOP**, C/EBP homologous protein, **TU**, tunicamycin, **TG**, thapsigargin, **GAPDH**, glyceraldehyde-3-phosphate dehydrogenase, **B2M**, beta-2-microglobulin, **RPL13A**, ribosomal protein L13a, **HPRT1**, hypoxanthine phosphoribosyltransferase 1, **BiP**, binding immunoglobulin protein, **BLOC1S1**, biogenesis of lysosome-related organelles complex 1 subunit 1, **PDI4**, Protein disulphide isomerase-like protein 4, **SYVN1**, E3 ubiquitin-protein ligase synoviolin, **HERPUD1**, Homocysteine-responsive endoplasmic reticulum-resident ubiquitin-like domain member 1 protein

## Abstract

The long-living naked mole-rat (NMR) shows negligible senescence and resistance to age-associated diseases. Recent evidence, based on protein-level assays, suggests that enhanced protein homeostasis machinery contributes to NMR stress-resistance and longevity. Here, we develop NMR-specific, transcriptional assays for measuring the unfolded protein response (UPR), a component of ER proteostasis. By varying doses and response times of pharmacological ER stressors applied to NMR kidney fibroblasts, we probe the NMR UPR in detail, demonstrating that NMR fibroblasts have a higher UPR activation threshold compared to mouse fibroblasts under mild ER-stress induction; whereas temporal analysis reveals that severe ER-stress induction results in no comparative differences. Probing NMR UPR activation with our robust assays may lead to insights into the proteostasis and ageing relationship.

## Highlights

• Healthy ageing in the naked mole-rat (NMR) may be linked to a robust proteostasis.
• Currently, NMR studies heavily rely on protein-level analysis of proteostasis.
• We report transcriptional assays probing the NMRs unfolded protein response (UPR).
• Distinct responses in NMR UPR observed between mild and severe ER stress induction.
• Our assays allow temporal changes of UPR markers in NMR fibroblasts to be monitored.

## 1 Introduction

The naked mole-rat (NMR; *Heterocephalus glaber*) is the longest-living rodent and shows negligible senescence over 20 years [1]. It has a maximal lifespan of over 30 years, five times longer than expected allometrically based on its body mass, while a similar-sized laboratory mouse (*Mus musculus*) lives up to 4 years [2]. NMRs exhibit no significant age-related changes in fertility, basal metabolism, cardiovascular and gastrointestinal functions and are highly resistant to cancer and neurodegenerative disorders [2,3]. Accumulating evidence suggests that the maintenance of robust proteostasis protects NMRs from various forms of stress, preserves the functionality of the proteome and contributes to healthy longevity [4]. Studies have shown that compared to shorter-lived rodents, NMRs possess increased proteasome activity and elevated basal levels of autophagy and heat-shock proteins [5,6]. However, these results are based predominantly on protein-level analyses, and our knowledge is limited about proteotoxic stress responses modulated at the transcriptional level in the NMR, including the unfolded protein response (UPR).

The UPR is a collection of pathways activated by misfolded proteins in the ER or perturbations of ER membrane lipids [7,8]. The mammalian UPR is defined by three transducers: inositol-requiring enzyme 1 (IRE1), protein kinase R-like ER kinase (PERK) and activating transcription factor 6 (ATF6), which initiate downstream programmes to restore proteostasis or trigger apoptosis [9]. Activated IRE1 excises an intron from the *Xbp1* mRNA, which produces spliced X-box-binding protein 1 (XBP1s) that upregulates ER chaperones and genes involved in ER-associated degradation (ERAD) and lipid synthesis [9]. Activated IRE1 also cleaves ER-localised mRNAs by regulated IRE1-dependent decay (RIDD) to alleviate the protein-folding stress in the ER [10]. Activated PERK phosphorylates the α subunit of translation initiator factor 2 (eIF2α), which inhibits protein synthesis but preferentially translates the activating transcription factor 4 (ATF4) [9]. Under prolonged ER stress, ATF4 induces expression of C/EBP homologous protein (CHOP), which upregulates pro-apoptotic proteins and leads to cell death [11]. Activated ATF6 is translocated from the ER to the Golgi apparatus where it is cleaved to release a fragment that induces expression of a range of UPR targets including protein disulphide isomerases and ER chaperones such as binding immunoglobulin protein (BiP) [9].

Involvement of the UPR is evident in the onset and progression of ageing and many age-associated diseases including cancer, metabolic diseases and neurodegenerative disorders [12]. However, irreconcilable results from different disease models have confounded the roles of the UPR [13]. As experimental manipulations of shorter-lived species may not fully model pathogenesis, increasing attention towards long-lived species that are naturally resistant to age-associated diseases is needed to fully understand the UPR [2,13]. Previous studies showed that NMR skin fibroblasts were surprisingly more sensitive to tunicamycin (TU) and thapsigargin (TG) than mouse counterparts, but the mechanisms were unclear [14]. TU induces ER stress by inhibiting N-glycosylation of newly synthesised proteins; TG depletes Ca^2+^ from the ER by blocking the sarcoplasmic and ER Ca^2+^-ATPase [15,16]. It is therefore of great interest to probe the UPR of the NMR in molecular detail, although this can be challenging as much of the NMR genome has not been annotated extensively [17].

In this study, we reported the first assays to examine the UPR of the NMR at the transcriptional level. We highlighted differences between the UPR of fibroblasts derived from NMR and mouse kidneys in response to *in vitro* ER stress. We determined that although no significant difference was observed in NMR and mouse fibroblasts under severe ER stress, a notably higher threshold of pharmacologically-induced UPR activation was identified in the NMR fibroblasts under mild ER stress.

## 2 Materials and methods

### 2.1 Animals

All experiments were conducted in accordance with the United Kingdom Animal (Scientific Procedures) Act 1986 Amendment Regulations 2012 under Project Licenses (70/7705 and P7EBFC1B1) granted to E. St. J. S. by the Home Office; the University of Cambridge Animal Welfare Ethical Review Body also approved procedures. Young adult NMRs (one female and four males, 0.5–2 years old) and C57BL/6J mice (males, 10–14 weeks old) were used in this study. Mice were conventionally housed with nesting material and a red plastic shelter in temperature-controlled rooms (21 °C) with a 12 h light/dark cycle and access to food and water *ad libitum*. Naked mole-rats were bred in-house and maintained in an inter-connected network of cages in a humidified (∼55 %) temperature-controlled room (28 °C) with red lighting (08:00-16:00) and had access to food *ad libitum*. In addition, a heat cable provided extra warmth under 2-3 cages/colony. Mice were humanely killed by cervical dislocation of the neck and cessation of circulation, whereas naked mole-rats were killed by CO_2_ exposure followed by decapitation.

### 2.2 Primary fibroblast isolation and culture

Animals were sacrificed and kidneys were harvested and incubated in an enzymatic digestion solution: 10 mg/mL collagenase (Roche), 1000 U/mL hyaluronidase (Sigma) in high-glucose DMEM (Thermo Fisher Scientific) at 37 °C for 30–60 min in a 5% CO_2_ incubator. Samples were then pelleted by centrifugation (500 g, 5 min) and resuspended in the culture medium: high-glucose DMEM, 15% fetal bovine serum (Sigma), 100 U/ml penicillin and 100 μg/mL streptomycin (Sigma), 1X MEM non-essential amino acids (Thermo Fisher Scientific), 1 mM sodium pyruvate (Thermo Fisher Scientific) and 100 μg/mL Primocin (InvivoGen). Cell suspensions were passed through a Falcon 70 μm cell strainer (Fisher Scientific) and seeded in a T25 culture flask. NMR cells were cultured at 32 °C in a 5% CO_2_, 3% O_2_ incubator; mouse cells were kept at 37 °C in a 5% CO_2_ incubator in air. Fibroblasts started to form colonies one week after incubation. Cells were subcultured in a T75 flask upon reaching 80% confluency. All fibroblasts were used within the first 5 passages. Cells were plated in six-well dishes (1.5 × 10^5^ cells/well) and incubated overnight before the addition of TU or TG (Cell Signaling Technology).

### 2.3 RNA extraction

Total RNA was isolated using the RNeasy Micro Plus Kit (Qiagen) and evaluated using a NanoDrop 2000 spectrophotometer (Thermo Fisher Scientific). RNA was mixed with the 2X RNA loading dye (Thermo Fisher Scientific) and visualised on a 1% agarose gel to confirm integrity after staining with SYBR Safe DNA gel stain (Thermo Fisher Scientific).

### 2.4 Xbp1 splicing assay

Reverse transcription of 1 μg RNA to cDNA was done with the ImProm-II™ Reverse Transcription System (Promega). PCR was performed to amplify *Xbp1s* and *Xbp1u* using cDNA. Mouse *Xbp1* primers were reported previously [18]; NMR *Xbp*1 primers were 5′-gaaccaggaattaaggatgcg-3′ and 5′-atccatggggagatgttctg-3’. The PCR products were separated by DNA electrophoresis on a 6% TBE gel (Thermo Fisher Scientific) and quantified with ImageJ to determine the *Xbp1s*-to-*Xbp1u* ratio.

### 2.5 Quantitative PCR

qPCR was performed using the StepOnePlus Real-Time PCR System (Applied Biosystems) and Powerup SYBR Green Master Mix (Thermo Fisher Scientific). For each primer pair, a standard curve was established with 5-fold serial dilutions of cDNA; primer efficiency was calculated from each standard curve [19]. Reference genes for each species/condition were selected using geNorm [20]. Mouse primers were purchased from OriGene (Rockville, USA). NMR primers were designed and listed in Table S1. Each qPCR reaction was performed in a 20 μl total volume with 10 ng cDNA and 200 nM primers under the conditions: 95 °C for 10 min, followed by 45 cycles of 95 °C for 15 s and 55 °C for 1 min. Each sample was analysed in duplicate. A melting curve was generated at the end of amplification. Relative expression levels of target genes were calculated by comparative C_T_ method [19].

### 2.6 Ca^2+^-imaging

Cells plated in 20 mm dishes (Thermo Fisher) were incubated in 10 μM Fluo-4 AM (Invitrogen) for 30 min at 21 °C. Dishes were then washed with extracellular solution (ECS), containing (in mM): NaCl (140), KCl (4), MgCl_2_ (1), CaCl_2_ (2), glucose (4) and HEPES (10) adjusted to pH 7.4 with NaOH, and imaged under an inverted Nikon Eclipse Ti microscope. Fluo-4 fluorescence was measured using an excitation wavelength of 470 nm LED (Cairn Research) and captured with a Zyla cSMOS camera (Andor) at 1 Hz with a 250 ms exposure time using Micro-Manager software (v1.4; NIH). A gravity-driven 12-barrel perfusion system was used to perfuse solutions in this system. During imaging, ECS was perfused for 10 s to establish the baseline, then TG (5, 50, 250 nM serially diluted in ECS) was perfused for 30 s. Each TG concentration was tested in separate culture dishes; 4 min after which ionomycin (10 μM, Cayman Chemicals) was applied for 10 s as a positive control in each dish. For quantifying increases in intracellular Ca^2+^, mean grey values of cells were extracted from manually drawn regions of interest (ROIs) in ImageJ. These values were then fed into a custom-made R-toolbox (https://github.com/amapruns/Calcium-Imaging-Analysis-with-R.git) to compute the proportion of cells responding to each concentration of TG and their corresponding magnitude.

### 2.7 Cell viability assay

NMR and mouse cells were plated in 96-well tissue culture plates (5 × 10^4^ cells/well). After overnight incubation, cells were treated with vehicle (DMSO), 0.1% Triton X-100 (Thermo Fisher Scientific), TU at 0.1, 1 and 5 μg/mL or TG at 5, 50 and 500 nM for 24 h. The percentage of viable cells in culture was then determined by CellTiter-Glo Luminescent Cell Viability Assay (Promega).

### 2.8 Caspase-Glo 3/7 assay

NMR cells were plated in 96-well tissue culture plates (5 × 10^4^ cells/well). After overnight incubation, cells were treated with vehicle (DMSO), 5 μM staurosporine (diluted in DMSO from 2 mM stock; Sigma), TU at 0.1, 1 and 5 μg/mL or TG at 5, 50 and 500 nM for 24 h. The percentage of apoptotic cells in culture was then determined by the Caspase-Glo 3/7 assay (Promega).

### 2.9 Statistical analysis

For analyse within the species (NMR or mouse), results from *Xbp1* splicing, RT-qPCR, cell viability and caspase assays under TU- or TG-treated conditions were compared with results from untreated controls using paired t-tests. For inter-species comparison (NMR versus mouse primary kidney fibroblasts), differences in the *Xbp1s*-to-*Xbp1u* ratio, gene expression level and cell viability were tested by two-way ANOVA tests and Sidak’s multiple comparisons tests. All statistical analyses were performed using Prism GraphPad 8.

## 3 Results

### 3.1 NMR kidney fibroblasts have a higher IRE1 activation threshold

**Fig. 1:**
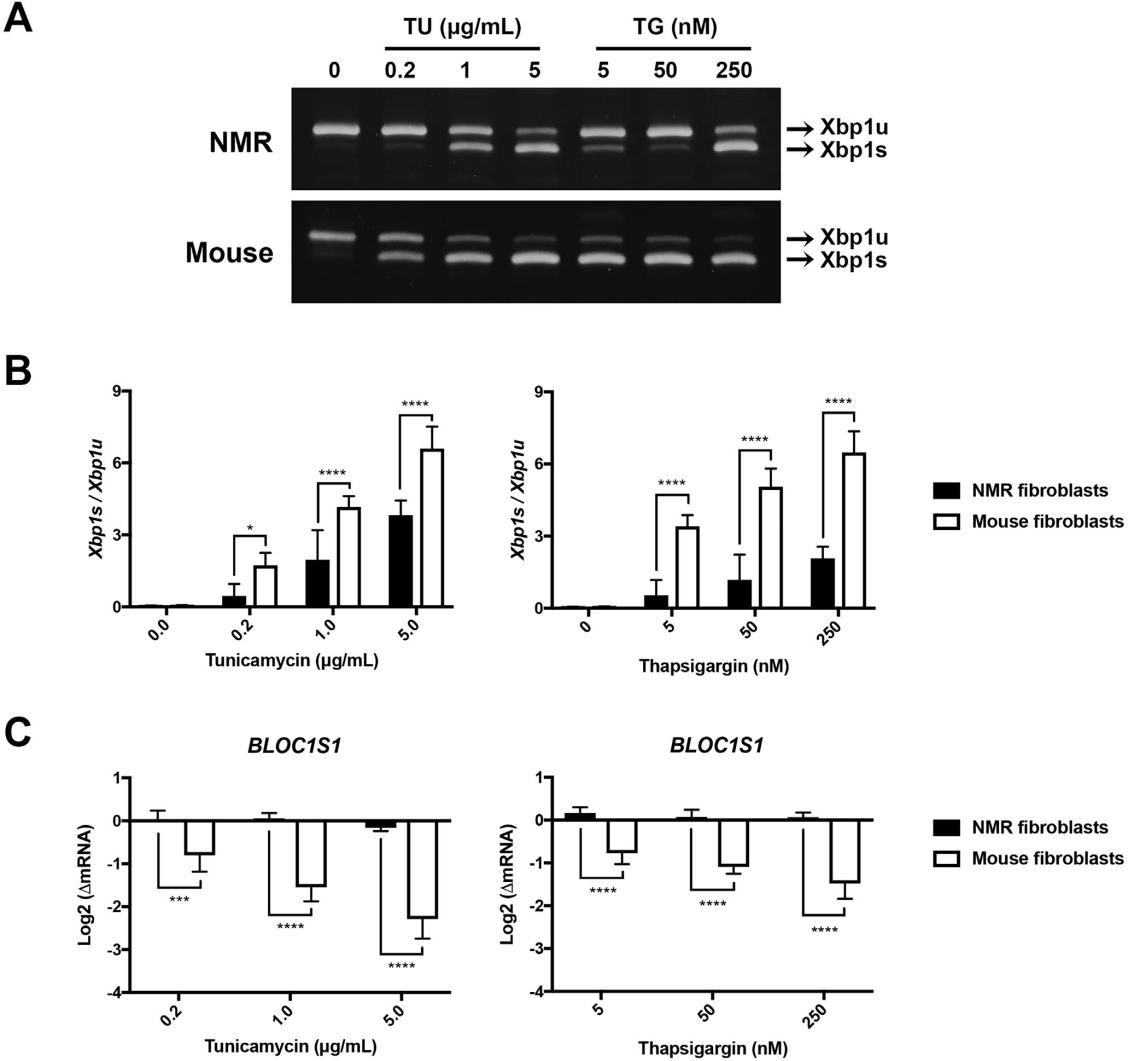
*Xbp1* splicing and RIDD degradation in the NMR and mouse kidney fibroblasts in response to TU (6 h) and TG (4 h). (A) Representative images of the RT-PCR products of *Xbp1s* and *Xbp1u* on 6% TBE gels. The graphs in (B) show the mean ± S.D. of *Xbp1s*-to-*Xbp1u* ratios in the NMR and mouse fibroblasts as a response to increasing concentrations of TU (left) or TG (right). n = 5 pairs; ∗*P* < 0.05; ∗∗∗∗*P* < 0.0001; two-way ANOVA tests, Sidak’s multiple comparisons tests. (C) *Bloc1s1* degradation measured by RT-qPCR. Data were presented as mean ± S.D. of log-transformed mRNA fold change compared to the basal-level expression in untreated controls. n = 5 pairs; ∗*P* < 0.05, ∗∗∗*P* < 0.001, ∗∗∗∗*P* < 0.0001; two-way ANOVA tests, Sidak’s multiple comparisons tests.

We first developed a *Xbp1* splicing assay in NMR kidney fibroblasts. The ratio of spliced *Xbp1* (*Xbp1s*) to unspliced *Xbp1* mRNA (*Xbp1u*) is a proximal reporter for IRE1 activation [11]. A dose-dependent titration determined that the *Xbp1* splicing occurred in the NMR fibroblasts after 6 hr-treatment of 1 μg/mL TU (∗*P* = 0.0240; n = 5) or 4 hr-treatment of 50 nM TG (∗∗*P* = 0.0034; n = 5) (Fig. 1A and B, top). Using the same experimental conditions, we found that the *Xbp1* splicing was detected in mouse kidney fibroblasts at 0.2 μg/mL TU (∗∗*P* = 0.0022; n = 5) and 5 nM TG (∗∗∗∗*P* < 0.0001; n = 5) (Fig. 1A and B, bottom). No basal-level *Xbp1* splicing was present in either species. At all drug concentrations, the *Xbp1s-to-Xbp1u* ratios were considerably lower in the NMR fibroblasts, suggesting a lower level of the IRE1 activation.

We then optimised the qPCR assays in the NMR fibroblasts to measure changes of the UPR genes. Following the MIQE guideline, we validated the efficiency of all qPCR primers (Table S1) and identified the optimal reference genes using geNorm [20,21]. The ideal reference genes, which had the lowest average expression stability values (M) out of the four housekeeping genes (Table 1), were determined in NMR and mouse kidney fibroblasts (*Gapdh/Rpl13a* for TU and TG-treated NMR fibroblasts; *Rpl13a/Gapdh* and *Rpl13a/Hprt1* for TU- and TG-treated mouse fibroblasts, respectively).

**Table 1:** Reference gene expression stability evaluation in the NMR and mouse.

Species	Tunicamycin				Thapsigargin			
	Genes	M	Mean CV (%)^a^	Mean M^b^	Genes	M	Mean CV (%)^a^	Mean M^b^
Naked mole-rat	*Gapdh*	0.157	19.7%	0.178	*Gapdh*	0.233	31.9%	0.268
	*Rpl13a*	0.198			*Rpl13a*	0.302		
Mouse C57BL/6J	*Hprt1*	0.155	29.6%	0.161	*Rpl13a*	0.295	37.8%	0.305
	*Rpl13a*	0.167			*Gapdh*	0.315		

a Required Mean CV (%): <50% for heterogenous samples [20].

b Required Mean M: < 1 for heterogenous samples [20].

Using our established qPCR protocol, we probed changes of a prominent marker of RIDD, *Bloc1s1* [10]. Results showed *Bloc1s1* levels did not change at any given TU or TG dose in the NMR fibroblasts but decreased in the mouse fibroblasts at 0.2 μg/mL TU and 5 nM TG (Fig. 1C), suggesting a lower level of the IRE1-mediated RIDD in the NMR fibroblasts under mild ER stress.

### 3.2 Lower induction of the ER protein-folding machinery is coupled with less activation of pro-apoptotic signals in the NMR kidney fibroblasts in response to mild ER stress

**Fig. 2:**
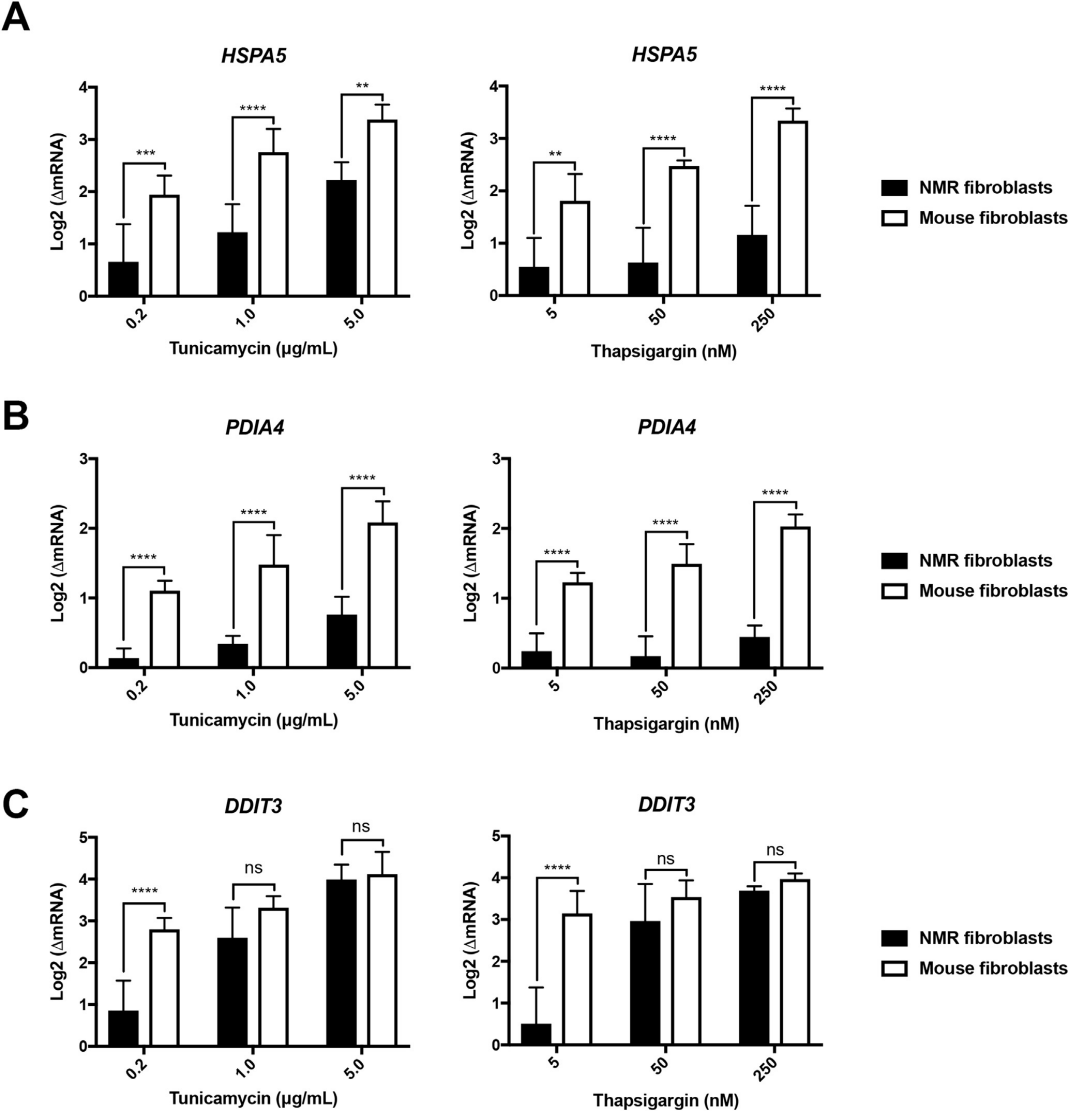
Induction of BiP/*Hspa5*, ERp72/*PDIA4* and CHOP/*Ddit3* in the NMR and mouse kidney fibroblasts in response to TU (6 h) and TG (4 h). All results were presented as mean ± S.D. of log-transformed mRNA fold change compared to the basal-level expression in untreated cells. n = 5 pairs; ns, *P* > 0.5, ∗∗*P* < 0.01, ∗∗∗*P* < 0.001, ∗∗∗∗*P* < 0.0001; two-way ANOVA tests, Sidak’s multiple comparisons tests.

We also evaluated changes of the ATF6- and PERK-regulated UPR genes in the NMR and mouse kidney fibroblasts. Levels of ATF6-regulated BiP/*Hspa5* and ERp72/*PDIA4* in the NMR fibroblasts were less than double at 1 μg/mL TU (∗∗*P* = 0.0069 for *Hspa5*, ^##^*P* = 0.0022 for *PDIA4*; n = 5) or 250 nM TG (∗∗*P* = 0.0090 for *Hspa5*, ^##^*P* = 0.0042 for *PDIA4*; n = 5), while *Hspa5* and *PDIA4* in the mouse fibroblasts were upregulated significantly by a 4-fold increase with treatments of 0.2 μg/mL TU or 5 nM TG, suggesting lower induction of the protein-folding machinery in the NMR fibroblasts (Fig. 2A and B).

RT-qPCR results also revealed a 7-fold and 17-fold increase of CHOP/*Ddit3* expression, a key marker of UPR-mediated apoptosis, from the basal level in the mouse fibroblasts in response to 0.2 μg/mL TU or 5 nM TG, respectively (Fig. 2C). In the NMR fibroblasts, a mild level of *Ddit3* induction was observed at 1 μg/mL TU (∗∗*P* = 0.0075; n = 5) or 50 nM TG (∗∗*P* = 0.0017; n = 5) but not statistically significant at 0.2 μg/mL TU (*P* = 0.0564; n = 5) or 5 nM TG (*P* = 0.2387; n = 5) (Fig. 2C). Interestingly, *Ddit3* induction within the NMR and mouse fibroblasts did not seem to differ much after exposure to higher doses (1 μg/mL TU (*P* = 0.1073; n = 5 pairs) or 50 nM TG (*P* = 0.3430; n = 5 pairs)) (Fig. 2C), which prompted us to trace the development of ER stress and the effects of severe ER stress in the NMR and mouse kidney fibroblasts.

### 3.3 NMR and mouse primary fibroblasts show similar levels of UPR activation in response to severe ER stress

**Fig. 3:**
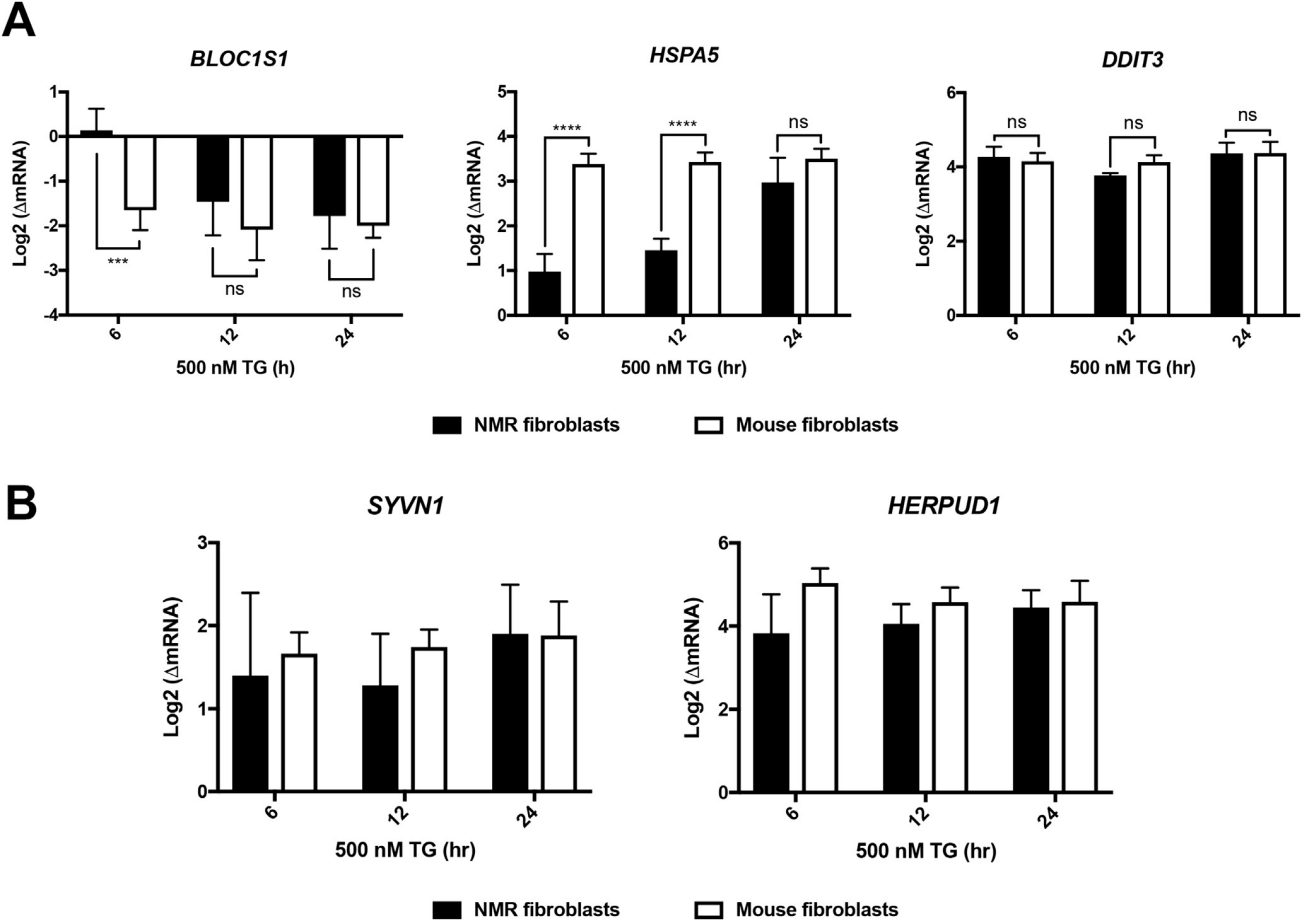
Changes of the UPR and ERAD markers in response to long-term treatment of 500 nM TG in the NMR and mouse kidney fibroblasts. (A) *Bloc1s1*, *Hspa5* and *Ddit3* and (B) *Svyn1* and *Herpud1*. All results were presented as mean ± S.D. of log-transformed mRNA fold change compared to the basal-level expression in untreated cells. n = 5 pairs; ns, *P* > 0.5, ∗∗*P* < 0.01, ∗∗∗*P* < 0.001, ∗∗∗∗*P* < 0.0001; two-way ANOVA tests, Sidak’s multiple comparisons tests.

When a higher dose of TG (500 nM) was applied, *Bloc1s1* degradation was observed in the NMR kidney fibroblasts after 12 hr-treatment (∗∗*P* = 0.0038; n = 5) and stabilised after 24-hr treatment at a 3-fold decrease, comparable to the level of *Bloc1s1* degradation in the mouse fibroblasts (Fig. 3A, left). Induction of *Hspa5* was significantly higher in the mouse fibroblasts after 6 hr- and 12 hr-treatments with 500 nM TG compared to the NMR fibroblasts, but the difference was diminished after 24-hr treatment (n = 5 pairs; *P* = 0.0560) (Fig. 3A, middle). Induction of *Ddit3* seemed to reach a plateau in both species after 6 hr-treatment, and no further upregulation was observed after 12 hr and 24 hr (Fig. 3A, right). These findings implied that NMR kidney fibroblasts were able to boost their transcriptional UPR levels to a similar extent as the mouse fibroblasts in response to severe ER stress.

We also examined the effects of high-dose TG on ERAD by probing the expression of *Syvn1* and *Herpud*, two ERAD components regulated by the UPR [22]. In NMR and mouse fibroblasts, induction of *Syvn1* and *Herpud1* was observed after 6hr-treatment, although the induction of *Syvn1* in the NMR fibroblasts was not statistically significant (*P* = 0.0864; n = 5) (Fig. 3B). Similar levels of *Syvn1*and *Herpud1* upregulation after 24 hr treatment suggest that severe ER stress did not result in differences of ERAD induction between the NMR and mouse kidney fibroblasts.

Finally, we determined the terminal effects of TU or TG on NMR and mouse kidney fibroblasts. Cell viability results showed that both species had similar levels of resistance to TU (up to 10 μg/mL) and TG (up to 500 nM) at any given dose (Fig. S1A), which were verified by a caspase 3/7 assay (Fig. S1B).

## 4 Discussion

Transcriptional analysis of the UPR has been established in many organisms but not in long-lived species. Widely-used antibody-based assays can be limited in physiological relevance as they require the induction of detrimental ER stress and have been reported to be problematic because of non-specific binding issues [23]. Here we developed RT-qPCR assays for NMRs with high sensitivity and reliability, which captured subtle changes of the UPR in response to low-dose, short-term treatments of TU and TG, allowing us to compare the NMR UPR under mild and severe ER stresses and to delineate species-specific mechanisms using the same experimental conditions.

We determined the appropriate reference genes for qPCR analysis in NMR and mouse kidney fibroblasts under TU and TG-treated conditions. Using the same geNORM algorithm, Schuhmacher and co-workers examined twelve housekeeping genes in the NMR nervous system and identified the best reference gene pair was *ACTINIB*/*EIF4A2* [24]. We tested four housekeeping genes and showed that they had more stable expression in NMR kidney fibroblasts, as indicated by M values, than in the nervous system, suggesting that the choice of reference genes is highly dependent on the tissue type and should be assessed stringently on a case-by-case basis.

We identified that NMR kidney fibroblasts had a higher threshold of UPR activation under mild ER stress, and such attenuated UPR was not due to differences in drug uptake between the NMR and mouse fibroblasts, as suggested by Ca^2+^ imaging results (Fig. S1). It was reported previously that a long-lived *daf*-2(−) strain of *C. elegans* showed a similar phenotype of lower *Xbp1* splicing but strong resistance to ER stress, which was attributed to the upregulation of a new set of genes involved in ER proteostasis by XBP1 and DAF-16 [25]. A broader analysis of the transcriptional profile can be applied to understand the effects of such an interplay in the NMR. Snell dwarf mouse fibroblasts also showed diminishing Xbp1 splicing but were sensitive to ER stress due to enhanced pro-apoptotic signalling including CHOP, which was not observed in the NMR kidney fibroblasts [26]. Lower induction of ER chaperones in the NMR kidney fibroblasts suggested less compensation for the protein-folding capacity in response to mild ER stress, likely as a result of higher basal-levels of chaperones and/or a more stable proteome in the NMR [4,6].

Salmon and colleagues reported sensitivity of NMR skin fibroblasts to ER stressors compared to mouse fibroblasts [14]. However, we found that NMR and mouse kidney fibroblasts showed similar levels of the UPR activation under severe ER stress and resistance to a broad dose range of TU and TG. These conflicting results might be due to differences of the experimental conditions where we minimised the introduction of additional stresses by keeping the NMR cells in a 32 °C hypoxic environment of 3% O_2_, which has been shown to enable their optimum growth, and by eliminating the serum-starvation step prior to TU or TG treatment suggested by Salmon et al. [14]. Discrepancies of these results may also reflect the differences between cells from different organs. Further studies can therefore be performed to investigate tissue or cell-type-specific UPR in the NMR, for example, in pancreatic β cells that are constantly challenged by ER stress.

This work constitutes the first investigation of the UPR in the NMR at the transcriptional level and shows insights into the differences of the UPR mechanisms in NMR and mouse kidney fibroblasts. Understanding the complex roles of the UPR is daunting, particularly in emerging species where molecular tools are limited. Our assays can be easily modified to explore expression of a range of genes in the UPR and other pathways in the NMR. With discoveries of drugs that modulate individual UPR branches, more studies can be conducted to elucidate detailed mechanisms of the UPR in the NMR, thereby promoting our understanding of the relationship between proteostasis and ageing.

## Funding sources

Z.D. is supported by a Herchel Smith PhD Research Scholarship and a Rosetrees Trust PhD Studentship. S.C. was supported by a Gates Cambridge Trust scholarship. The work was supported, in part, by the Centre for Misfolding Diseases (Z.D., C.M.D., J.R.K.), the 10.13039/100010269Wellcome Trust (094425/Z/10/Z; J.R.K., C.M.D.) and the 10.13039/501100000289Cancer Research UK Multidisciplinary Project Award (C56829/A22053; E.S.S.). Funding agencies were not involved in any aspects of the planning, experiments, data collection and analysis, or writing and editing of the manuscript. There are no conflicts of interest to disclose.

## Declaration of competing interest

The authors declare that they have no known competing financial interests or personal relationships that could have appeared to influence the work reported in this paper.
