# Supplementary material for: Probing the unfolded protein response in long-lived naked mole-rats
Source: Biochem Biophys Res Commun. 2020 Sep 3;529(4):1151–7. doi: 10.1016/j.bbrc.2020.06.118 (PMC7453385; doi:10.1016/j.bbrc.2020.06.118)
Supplement: Multimedia component 1 [file mmc1.pdf]

Supplementary Information

Probing the unfolded protein response in long-lived naked mole-rats

Zhen Du[1,2], Sampurna Chakrabarti[1], Yavuz Kulaberoglu[1,3], Ewan St. John Smith[1], Christopher M. Dobson[2,+], Laura S. Itzhaki[1,*] and Janet R. Kumita[1,2,*]

[1]Department of Pharmacology, University of Cambridge, Tennis Court Rd., Cambridge, CB2 1PD, UK; [2]Centre for Misfolding Diseases, Department of Chemistry, University of Cambridge, Lensfield Rd., Cambridge, CB2 1EW, UK; [3]UCL Institute of Healthy Ageing, Darwin Building, 104 Gower St, Bloomsbury, London, WC1E 6AD, UK

Table S1. NMR primers used in this study. Primers for qPCR assays were designed based on the published NMR genome published [1]. The efficiency of each primer pair was confirmed by standard curve method to be within 90% to 110% [2]. The specificity of each primer set was also validated by a melting curve analysis in which a single melting temperature peak was observed for each qPCR reaction.

Gene	Primers	Efficiency
Gapdh	5'-cctgccgcctggagaaa-3'[3]<br>5'-cgcctgcttcaccacctt-3'[3]	95%
B2m	5'-gcataccaacttcaccccca-3'<br>5'-ccaggctcacacaggaagtg-3'	99%
Hprt1	5'-ccaaagcaggaaaaacgaca-3'[4]<br>5'-catccagagggagaaaggttc-3'[4]	103%
Rpl13a	5'-tgaggttggctggaagtatcag-3'<br>5'-agtggatcttggccttctcctt-3'	96%
Hspa5	5'-tcttgcgtcggagtgttcaa-3'<br>5'-cgtgttctcggggttagagg-3'	94%
Ddit3	5'-gagctggaagcctggtatga-3'<br>5'-attctcttcgtttccaggggg-3'	106%
Bloc1s1	5'-gagctggaagcctggtatga-3'<br>5'-attctcttcgtttccaggggg-3'	106%
PDIA4	5'-cgtgctgagtgttgagggt-3'<br>5'-atcgttggaggtcttgcggt-3'	100%
Syvn1	5'-aggtgatgggcaaggtgttc-3'<br>5'-cccgaaaaacggtgaaggc-3'	106%
Herpud1	5' -ccaaagcaggaaaaacgaca- 3'<br>5' -catccagagggagaaaggttc- 3'	99%

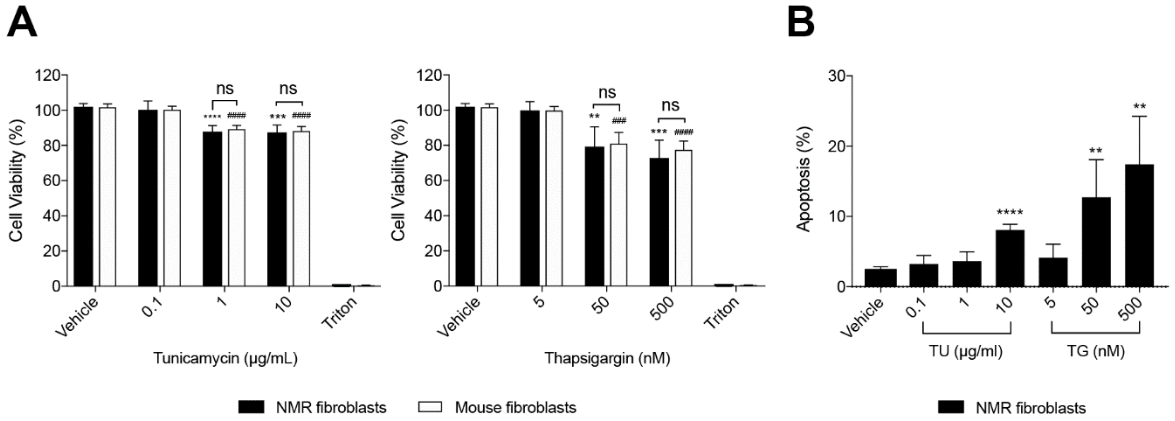

Figure S1. Percentage of the viable NMR and mouse primary kidney fibroblasts after exposure to ER stressors (A). Cells were left untreated or treated with vehicle (DMSO), 0.1% Triton, TU and TG for 24 hr and evaluated for viability by Promega CellTiter-Glo Luminescent Cell Viability Assay. Results were presented as mean ± S.D. of the percentage survival (%) compared to the untreated control. Significant decrease in cell survival was observed in the NMR primary kidney fibroblasts (n=5; **P*<0.05, ***P*<0.01, ****P*<0.001; paired t-tests) and mouse primary kidney fibroblasts (n=5; $^{\#\#}P<0.01$, $^{\#\#\#}P<0.001$; paired t-tests) treated with ≥ 1 TU µg/mL or ≥ 50 nM TG in reference to vehicle controls. No significant difference was observed in the NMR and mouse primary kidney fibroblasts under these conditions (n=5 pairs; ns, *P*>0.5; two-way ANOVA tests; Sidak's multiple comparisons tests). Percentage of apoptosis in the NMR primary kidney fibroblasts in response to TU and TG at various doses (B). Cells were left untreated or treated with vehicle (DMSO), 5 µM staurosporine, TU and TG for 24 hr and evaluated for apoptosis by Caspase-Glo 3/7 Assay. Results were presented as mean ± S.D. of the percentage apoptosis (%) compared to the staurosporine control. Significant cell apoptosis was observed at 5 µg/mL TU (n=5; *****P*<0.0001; paired t-test), 50 nM TG (n=5; **P*=0.0119; paired t-test) and 500 nM TG (n=5; ***P*=0.0080; paired t-test).

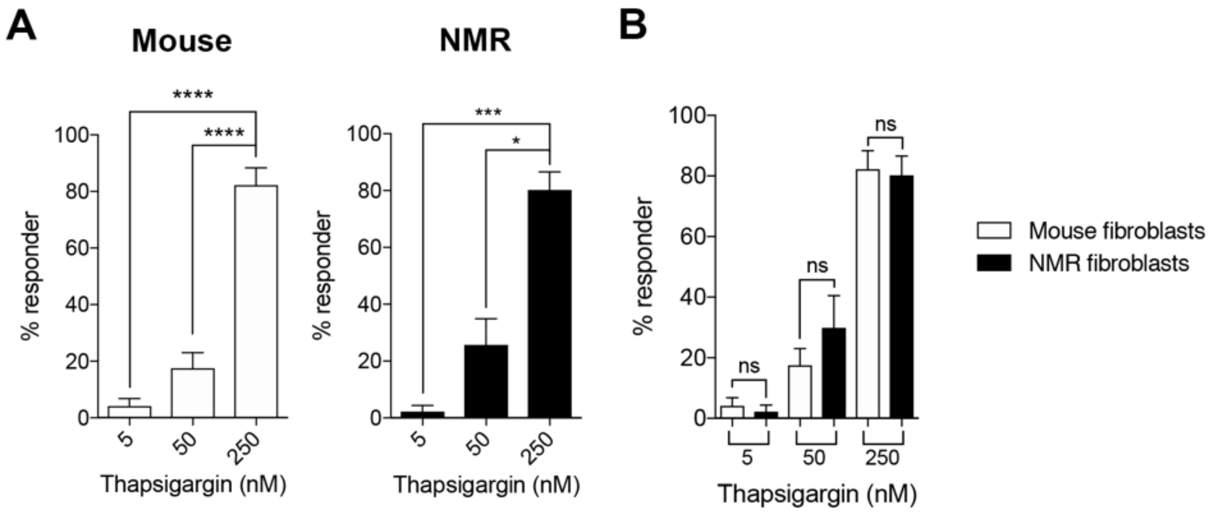

Figure S2. The efficacy of TG on the NMR and mouse primary kidney fibroblasts was measured by Ca^{2+} imaging. Data were obtained from 5 mice and 5 NMRs' >350 cells were imaged in each condition. TG increased $[Ca^{2+}]$ in mouse (A, left) and NMR (A, right) primary kidney fibroblasts in a similar dose-dependent manner (n=5; * $P<0.05$, *** $P<0.001$; paired t-tests). Similar proportions of mouse and NMR primary kidney fibroblasts responded to TG at all doses (B, n=5 pairs; ns, $P>0.5$; two-way ANOVA tests; Sidak's multiple comparisons tests). Ionomycin was used as a positive control for data normalisation.

Reference

[1] E.B. Kim, X. Fang, A.A. Fushan, *et al*., Genome sequencing reveals insights into physiology and longevity of the naked mole rat, Nature. 479 (2011) 223–227. https://doi.org/10.1038/nature10533.

[2] T.D. Schmittgen, K.J. Livak, Analyzing real-time PCR data by the comparative CT method, Nat. Protoc. 3 (2008) 1101–1108. https://doi.org/10.1038/nprot.2008.73.

[3] S. Zhao, L. Lin, G. Kan, *et al.*, High autophagy in the naked mole rat may play a significant role in maintaining good health, Cell. Physiol. Biochem. 33 (2014) 321–332. https://doi.org/10.1159/000356672.

[4] E.S.J. Smith, Omerbašić, S.G. Lechner, G. *et al.*, The molecular basis of acid insensitivity in the African naked mole-rat, Science. 334 (2011) 1557–1560. https://doi.org/10.1126/science.1213760.
